# Neglected Men's Wards

**Published:** 1889-04-20

**Authors:** 


### NEGLECTED MEN'S WARDS.

I saw a letter in the *Times* and also in *The Hospital* of March 30, headed "The Court and the Hospital," on the subject of ladies giving their bouquets to some children's hospital or wards. Now, sir, there is no one loves children more than I do, but I maintain there is sadly too much spent on them in hospitals, at any rate too much sentiment. A rag doll or a few buttercups or daisies would be quite as much appreciated as the most expensive toys or the most magnificent bouquets. I was a nurse, and afterwards a "sister," in two large London hospitals, and also in a large one in Liverpool for many years, so I speak from experience; and I always found the "bread-winners'" (men's) wards particularly were the least thought of or decorated, and I know the men appreciate the beautiful quite as much, if not more, than their wives, sisters, and poor little children, but they generally get—in large hospitals at least—what is left. I say send the bouquets to the men's wards, and it will give them something to talk about.

E. H. A.

March 31, 1889.

